# How to make mental health services more youth‐friendly? A Delphi study involving young adults, parents and professionals

**DOI:** 10.1111/hex.13832

**Published:** 2023-08-22

**Authors:** Eline Wittevrongel, Ruud van Winkel, Maarten Jackers, Laura Colman, Melina Versyck, Eline Camp, Geert Everaert, May Vrijens, Dieter Baeyens, Marina Danckaerts

**Affiliations:** ^1^ Department of Neurosciences, Research Group Psychiatry, Center for Clinical Psychiatry KU Leuven Leuven Belgium; ^2^ University Psychiatric Center (UPC) KU Leuven Leuven Belgium; ^3^ Faculty of Medicine KU Leuven Leuven Belgium; ^4^ mArquee Multiversum Psychiatric Hospital Antwerpen Belgium; ^5^ Neuro‐Psychiatric Clinic Saint Joseph V.Z.W. Pittem Belgium; ^6^ Asster Psychiatric Hospital Sint‐Truiden Belgium; ^7^ Faculty of Psychology and Educational Sciences, Parenting and Special Education KU Leuven Leuven Belgium; ^8^ Department of Neurosciences, Research Group Psychiatry, Center for Developmental Psychiatry KU Leuven Leuven Belgium

**Keywords:** Delphi study, mental health, patient‐oriented research, service design, youth

## Abstract

**Introduction:**

Although youth‐friendly service characteristics have been previously identified, consensus among a representative group of stakeholders about which of these characteristics are truly relevant to the youth‐friendliness of services is currently lacking. In our study, young adults, parents and professionals were consulted on this topic to reveal existing (dis)agreement. In addition, (dis)agreement on feasibility for implementation in clinical practice was also assessed.

**Methods:**

A mixed‐method Delphi approach was used with three online questionnaire rounds and a physical meeting. Young adults (18–26 years) and parents were part of a public panel and professionals were allocated to the professional panel. In the rounds, participants were asked to rate the importance and feasibility of each item. Subsequently, the percentage agreement (% of participants giving a score of 7 or above on a 9‐point Likert scale) within and across panels was calculated. Consensus was assumed to have been reached when at least 70% agreement was achieved. A thematic analysis of the qualitative data, obtained in the rounds and the physical meeting, was performed to identify overarching themes and characteristics of relevance to the youth‐friendliness of services.

**Results:**

For 65% of the items included in the Delphi questionnaire, consensus on importance was reached within both panels. Participants showed more insecurity about the feasibility of these items, however. Our thematic analysis revealed reasons for disagreement between and within the panels.

**Conclusions:**

Our study revealed substantial between‐ and within‐panel agreement on youth‐friendly service characteristics. We recommend that the items for which consensus was reached should be used as a checklist in terms of youth mental health service development, design and delivery. The characteristics for which there was disagreement between and within the panels should inspire an ongoing trialogue between young adults, parents and professionals both on the individual level and the service level.

**Patient or Public Contribution:**

In this study, (parents of) young adults with lived experience were included as experts, including one of the coauthors. This coauthor contributed to the manuscript by having a final say about the included quotes.

## 1 INTRODUCTION

A first‐episode mental disorder most frequently emerges before the age of 25.^1^ Eating disorders, obsessive‐compulsive disorders, alcohol and cannabis use disorders, schizophrenia, personality disorders and panic disorders usually begin in adolescence and young adulthood. Additionally, even early presentations of mental disorders that are still below the threshold for traditional diagnostic categories can be associated with distress, functional impairment and diminished quality of life in this age range.^2^, ^3^

Although prevalence rates of mental disorders are high in young adults, a large proportion does not find their way into mental health care.^4^ Twelve‐month treatment rates are very low with estimates of about 20%–30% for mental disorders.^5^, ^6^ Several barriers are at play, such as stigma, a lack of knowledge about mental health and mental health services, a preference for self‐reliance, feeling unworthy of help, concerns about confidentiality and fear of a negative response after disclosure.^7^, ^8^, ^9^, ^10^, ^11^, ^12^ Even if these internal barriers are overcome, pathways to care are complex due to multiple help‐seeking contacts (with general practitioners, emergency services, etc.) and long waitlists, sometimes resulting in a delay of appropriate care.^8^, ^13^

One of the guiding principles for youth mental health services, proposed by Hughes et al.,^14^ is that services should embody a youth‐friendly approach to service delivery. Inflexible hours, high costs, rigid age boundaries, unengaged staff and a plain décor can lead to a negative experience, which may result in disengagement.^15^ Services being youth‐friendly, on the other hand, might have a positive impact on service use and treatment engagement.^16^, ^17^ Previous studies have indeed found that the likelihood of help‐seeking increases when youth have positive past experiences with accessing and receiving mental health services.^8^, ^18^ In 2012, the World Health Organization (WHO) recognized the need for a framework for adolescent health care and stated that youth‐friendly services should be accessible, acceptable, equitable, appropriate and effective.^16^ This framework applies to health services in general, including physical care. More recently, Hawke et al. proposed a definition for mental health and substance use services specifically and identified a broad array of characteristics, based on a review of the literature and stakeholder consultations. Although eight caregivers (i.e., members of a young person's social network) and 19 service providers took part in the study, only five young adults were consulted.^17^

While characteristics have been previously identified, information on consensus among a representative group of stakeholders about which of these characteristics are truly relevant to the youth‐friendliness of services is currently lacking. Up until now, this has resulted in great variability across services in which characteristics are implemented and how: for instance, peer support is not always offered and only a few services involve young people in the design of the service area.^19^, ^20^, ^21^ Moreover, especially young people should be heard given that involving them in service development, design and delivery helps to improve access and increases engagement in and satisfaction with services.^17^ Therefore, in our study, the first aim was to investigate to what extent different stakeholder groups, that is, young people, parents and professionals, (dis)agree about the relevance of implementing previously identified characteristics to the youth‐friendliness of services.

Second, given that the development of new services or substantial changes within existing services might prove challenging if met with resistance due to limited resources in mental health care or scepticism in the field,^4^ feasibility of these characteristics should also be considered. Prioritizing the most important characteristics that are also deemed highly feasible for implementation might prove to be quick wins for services to improve upon their youth‐friendliness. Therefore, in our study (dis)agreement on feasibility for clinical practice was also assessed.

## 2 METHODS

### 2.1 Study design

A mixed‐method Delphi approach was used. A Delphi study is an iterative multistage process that consists of a series of ‘rounds’ in which stakeholders, usually referred to as experts, are asked to give their opinion using structured questionnaires with the aim of developing consensus on items related to a certain topic.^22^, ^23^, ^24^ This technique is regularly used in mental health research, and although experts in Delphi studies are mostly professionals, the voices of service users and their caregivers have been increasingly heard by inviting them to be part of the expert panel.^24^, ^25^, ^26^ The current study consisted of three phases: after development of the questionnaire (phase 1), three online questionnaire rounds (phase 2) and a physical consensus meeting took place (phase 3).

### 2.2 Delphi expert panel members

Participants were included in one of two panels: young people and parents were allocated to the public panel and professionals were part of the professional panel, as is common in Delphi studies with multiple stakeholders.^24^ This allowed us to explore consensus within and across panels. We sought to include young people (16–27 years) that had experience with care provision in specialized mental health services (Child and Adolescent Mental Health Services [CAMHS] and/or Adult Mental Health Services [AMHS]). Young people had to have received or be still receiving care from a specialized mental health service between the ages of 16 and 24, and not in a private practice. Parents of young people who fulfilled these criteria were also allowed to take part. Professionals (i.e., service managers, psychologists, psychiatrists, social workers, etc.) were required to have at least 3 years of work experience with young people and had to be active in social welfare services or mental health care.

**Figure 1:**
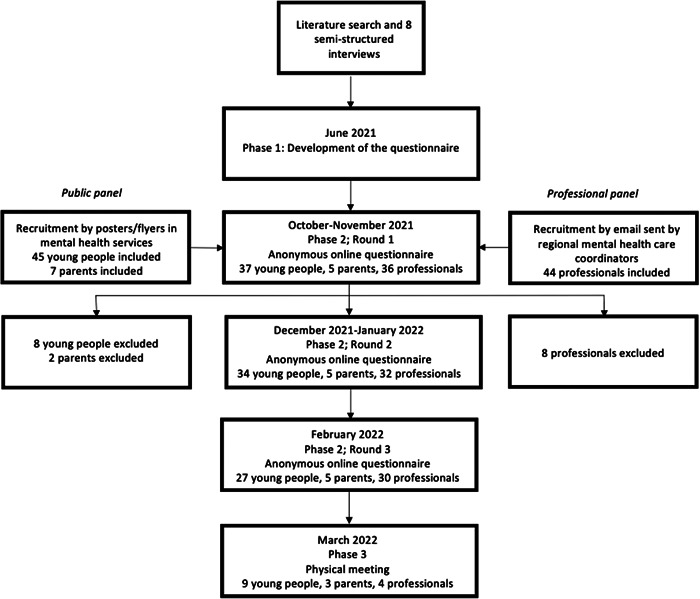
Overview of the Delphi study process including the number of participants per phase.

Over a period of 3 months, purposive sampling took place. We sent out an email with study information to regional mental health care coordinators that passed on the invitation to services working with young people. Professionals were encouraged to participate in the study and/or request a package of posters and flyers to recruit young people and parents within their own service. The inclusion criteria for the study were checked in a short online questionnaire. Figure 1 shows the number of participants in both panels at the time of inclusion. Eighteen professionals who fulfilled the inclusion criteria were informed they could not participate in the study to avoid numeric disbalance between the public panel and professional panel. Inclusion of professionals with extensive experience with young people (i.e., more hours of contact per week) was prioritized. Only young people who were older than 18 years could be included in the study given that recruited minors were not comfortable completing the additional administrative steps necessary to get explicit consent from a parent/guardian. Young adults received a gift card for every part of the study they had completed as compensation. Parents and professionals who completed the entire study received a voucher that granted free access to a study feedback event organized by the research group.

### 2.3 Phase 1: Delphi questionnaire

Based on the literature review by Hawke et al.,^17^ we developed a set of questions for a semistructured interview with eight youth mental health teams in Flanders. In this interview, we asked the participants to give concrete examples of how the identified youth‐friendly characteristics were already implemented in practice or how they would like to see them implemented. The examples were then formulated as items for the Delphi questionnaire. Before the start of the rounds, we asked two young adults with lived experience that did not participate in the study to review the questionnaire's feasibility in terms of length and ease of understanding. The final questionnaire consisted of 62 items pertaining to 25 characteristics mentioned by Hawke et al.^17^ (see Table 1 in the Supporting Information Appendices). REDCap (Research Electronic Data Capture), a secure web‐based software platform designed to support data capture for research studies, was used to develop and administer the questionnaire.^27^, ^28^

### 2.4 Phase 2: Delphi rounds

At the beginning of each round, participants received an invitation via email with a personal web link to the questionnaire. The rounds were open for completion during 21 days on average and reminder emails were sent every 5–6 days.

Completion of a round required panel members to rate each item in the questionnaire using a 9‐point Likert scale to indicate both their agreement with the importance of the item and its implementation feasibility separately. Participants were encouraged to also provide a commentary on why they considered the item (un)important using a feedback field, which resulted in completion of at least half of these fields in rounds 1 and 2.

Although there is no single definition of consensus for Delphi studies, the most common is percentage agreement, with 75% being the median threshold to define consensus.^29^ In studies that assess simultaneous agreement across multiple panels involving stakeholders with heterogeneous backgrounds (e.g., professionals, consumers, caregivers), a lower cut‐off for consensus is also accepted.^24^ Therefore, in our study, we defined consensus for the importance of an item as a score of 7 or above on the 9‐point Likert scale as endorsed by ≥70% of the panelists. The items for which consensus across the panels was reached were not readministered to the participants in the consecutive round(s).

In round 1 we also asked questions on demographics and experience with mental health care, specific to each stakeholder group (young adults, parents, professionals). Based on the feedback of the participants in round 1, the descriptions of 15 items were modified (see Table 2 in the Supporting Information Appendices) and 12 items were added to the questionnaire, resulting in the coverage of three additional characteristics described by Hawke et al.^17^ (see Table 1 in the Supporting Information Appendices).

For rounds 2 and 3 automated individualized questionnaires were prepared for each participant including the individual's rating for each item as well as the panel's median rating and a summary of the qualitative feedback covering the perspectives of both panels from the previous round. In round 2, we still allowed for differences between the panels by only showing participants the median scores of their own panel, while in round 3, we sought for consensus more by showing the median scores across both panels.

Partial completions were included in the analyses (round 1 = 3% of completions, round 2 = 11%, round 3 = 7%). Only participants who fully completed the questionnaire in round 1 were invited to participate in rounds 2 and 3. All data were collected between October 2021 and February 2022.

### 2.5 Phase 3: Physical meeting

All participants who completed round 1 were invited for a 3‐h physical meeting. We strived to have sufficient participants attending to conduct two focus groups in which all stakeholder groups were represented (for reasons of validity). In the first hour, the results from the three rounds were presented to the participants. Afterwards, in a focus group discussion, we asked the participants to look at the items that reached consensus in the public panel but not in the professional panel and vice versa. Participants were encouraged to come up with potential reasons for these discrepancies, reflecting on each panel's underlying motives. The meeting took place in March 2022 and concluded the study for the participants.

### 2.6 Statistical analysis

All analyses were performed using SPSS 28.^30^ The demographic data were analysed using descriptive statistics. For each item the percentage agreement on importance (i.e., the percentage of panelists who rated the item 7–9) and feasibility (if applicable) was calculated, within (i.e., in each panel separately) and across (i.e., pooling all panel data) the panels. We also determined the median, minimum, maximum and interquartile range for each item, both for agreement on importance and feasibility (if applicable).

### 2.7 Qualitative analysis

NVivo was used for qualitative data analysis.^31^ The qualitative data were thematically analysed using Braun and Clarke's process to identify overarching themes and subthemes, which we will refer to as ‘characteristics’.^32^ There were two bodies of data: the commentaries from the rounds and the transcript from the physical meeting. E. W. and M. J. discussed the themes and characteristics to ensure that they accurately captured the comments. For each characteristic, we further investigated why participants (dis)agreed on its relevance to the youth‐friendliness of services.

## 3 RESULTS

### 3.1 Response

Figure 1 shows the number of young adults, parents and professionals who participated in the consecutive rounds and the physical meeting. Overall, the response rate in the public panel for round 3 in comparison to round 1 was 76%. For the professional panel, this was 83%. Sixty‐two percent of the participants in the public panel and 78% of the professionals in the professional panel completed all the rounds. Finally, we had sufficient participants to conduct three focus groups during the physical meeting.

### 3.2 Participant characteristics

Most young adults participating in the study were female (89%). They had a mean age of 21.3 years (SD = 2.3) with a minimum and maximum age of 18 and 26, respectively. On a scale from 1 to 5 (1 = ‘bad’, 5 = ‘excellent’), young adults rated their mental health as being worse (median = 1.6, SE = 0.1) than their physical health (median = 2.7, SE = 0.1). Young adults who were still using mental health services were mostly satisfied with their care (median = 4.0 on a scale from 1 to 5, SE = 0.1).

All participating parents were female. Their young adult children were mostly still living with them (80%). Parents indicated to be satisfied with the care their children received (median = 4.0 on a scale from 1 to 5, SE = 0.6).

In the professional panel, the male‐to‐female ratio was more balanced (58% female). The professional panel was mixed in terms of professional group and type of work activity (see Table 1).

**Table 1:** Characteristics of participants in the panels in the first round.

Public panel	*N* (%)
*Young adults (n = 37)*	
Gender^a^	
Female	33 (89)
Male	4 (11)
Age, mean (SD)	21.4 (2.3)
Birthplace	
Belgium	34 (92)
Other	3 (8)
Living situation	
With parent(s)	21 (57)
Cohousing with friends	6 (16)
In a facility	7 (19)
Alone	3 (8)
Currently in education	
Secondary school	4 (11)
Vocational training	3 (8)
Bachelor's/master's degree	10 (27)
Adult learning initiatives	3 (8)
Currently working	
Yes	9 (24)
Diagnosis^b^	
Major depressive disorder	21 (57)
Posttraumatic stress disorder	9 (24)
Anxiety disorder	8 (22)
Personality disorder	8 (22)
Autism spectrum disorder	8 (22)
Multiple	21 (57)
Currently accessing mental health services	
Yes	34 (92)
Experience with mental health services	
Outpatient care	17 (46)
Inpatient care	6 (16)
Out‐ and inpatient care	14 (38)
*Parents (n = 5)*	
Gender	
Female	5 (100)
Age, mean (SD)	52.2 (6.5)
Relationship to child	
Biological mother	5 (100)
Currently working	
Yes	4 (80)
Child currently living with parent(s)	
Yes	4 (80)
Child's diagnosis^b^	
Major depressive disorder	3 (60)
Autism spectrum disorder	3 (60)
Multiple	4 (80)
Child currently accessing mental health services	5 (100)
Experience with mental health services	
Outpatient care	4 (80)
Inpatient care	1 (20)
*Professional panel (n = 36)*	
Gender	
Female	21 (58)
Male	15 (42)
Age, mean (SD)	42.8 (10.0)
Professional group	
Psychiatrist	5 (14)
Psychologist/therapist	15 (42)
Nurse/mental health worker	9 (25)
Social worker	3 (8)
Other	4 (11)
Type of work activity	
Outpatient care	10 (28)
Inpatient care	16 (44)
Youth support	7 (19)
Other	3 (8)
Years of experience, mean (SD)	17.4 (9.9)
Primary clinical population youth (16–24 years)	24 (67)
Followed/following postgraduate psychotherapy course	19 (53)

^a^ Female and male were the only identities reported.

^b^ Nonexhaustive list.

### 3.3 Quantitative results

#### 3.3.1 (Dis)agreement on importance

Forty‐seven items (65%) (see Table 2) reached consensus within both panels (round 1 = 25, round 2 = 15, round 3 = 7), while 20 items (28%) (see Table 3) only reached consensus in one panel (round 1 = 11, round 2 = 5, round 3 = 4). Of the latter, consensus was mainly reached within the professional panel (12 items), leaving eight items that were only endorsed within the public panel.

**Table 2:** Items with consensus on importance (70% or more endorsing a score of 7–9) within both panels, including % agreement for feasibility.

Number	Description of item upon consensus	Round	% Agreement importance		% Agreement feasibility^a^	
			Public	Professional	Public	Professional
*Stage: Initial contact*						
1.1	Community mental health services should fight stigma by collaborating with influencers.	3^b^	70	76	22	30
1.2	Community mental health services should make use of social media to gain more exposure.	2^b^	72	91	61	75
1.3	Services should have a folder or information brochure to inform YP and parents about how they operate.	2^b^	77	75	90	90
1.4	YP that have experience with the service can be asked to inform other YP about it, for example, by taking up a position as ‘ambassador’ for the service.	2	80	83	33	61
1.5	Services should allow YP to contact them themselves with a request for help.	1	78	97	66	79
1.6	Services should only ask for referrals if they are highly specialized, thus only offering help to a specific group.	3^b^	71	72	74	87
1.7	Services should strive to let the YP know shortly after putting in a request if they are eligible for getting help in the service.	2^c^	100	100	65	69
1.8	YP that don't know which service to get help from should be assisted by professionals in community services that direct them to ‘the right door’.	2^b^	95	100	/	/
1.9	Services should invite YP before the start of treatment so that YP can decide whether the service suits them.	1	95	91	75	89
1.10	Services that offer help to YP should be familiar with each other to facilitate referrals.	2^c^	92	97	56	77
1.11	Services should be located close to public transport.	1	90	100	39	38
1.12	Services should make sure that YP (and their parents) are welcomed by a staff member upon entry.	3^c^	74	97	68	77
1.13	Services should have free WIFI.	2^b^	84	83	87	79
1.14	Services should create a pleasant entrance or waiting area, for example, with youthful decoration, a water dispenser and easily accessible restrooms.	2^c^	87	93	81	76
1.15	Services should have a recreation area, for example, with a ping pong or pool table, karaoke.	1	78	77	78	63
1.16	Services should have a cozy communal area where YP can, for example, chat, play games or watch TV.	1	93	83	88	77
*Stage: Treatment*						
1.17	Services that offer help to YP should adapt their provision of care to the specific needs of YP.	2^b^	90	97	/	/
1.18	Developing a positive self‐image as well as discovering one's own talents should be an important focus in the treatment of YP.	1	95	94	/	/
1.19	Identity formation (What does the young person stand for? What does he/she/they find important in life?) should be an important focus in the treatment of YP.	1	100	100	/	/
1.20	Learning to become an autonomous individual and self‐management should be an important focus in the treatment of YP.	1	85	91	/	/
1.21	Learning how to cope with intense emotions and stress should be an important focus in the treatment of YP.	1	98	97	/	/
1.22	Learning how to identify and manage one's own emotional triggers should be an important focus in the treatment of YP.	1	88	97	/	/
1.23	Learning to be assertive and voice one's opinion should be an important focus in the treatment of YP.	1	88	89	/	/
1.24	Discovering your own limits (What is my comfort zone, stretch zone and panic zone?) should be an important focus in the treatment of YP.	2^b^	95	100	/	/
1.25	Services that offer help to YP must create a safe space where YP feel at ease.	1	95	94	/	/
1.26	Services should have a quiet room.	1	90	89	83	80
1.27	Services should involve volunteers in the care provision for YP, for example, for having a quick chat with the YP, organizing activities or practical support.	3	87	83	39	25
1.28	Services should strive for diversity in the team of care providers, for example, gender balance, ethnic diversity, LGBTQIA+ representation.	1	81	74	56	40
1.29	Healthcare providers should be careful not to be prejudiced based on information from previous care providers.	1	73	79	50	58
1.30	Services should prioritize the needs of YP over the needs of others in their network, for example, parents, external professionals.	1	90	82	73	63
1.31	Services should work with a care plan according to the goals of the YP themselves.	1	93	97	83	86
1.32	Services should allow YP to choose the format of the contacts between the young person and the healthcare provider, for example, online or face‐to‐face.	3^c^	83	76	68	67
1.33	When YP and/or their parents do not feel at ease with the healthcare provider, YP should be given the opportunity to switch providers within the service.	2^b^	76	70	43	57
1.34	Services should allow YP to contribute to the writing of their discharge letter, for example, add things or write the conclusion.	1	83	77	61	47
1.35	YP must be allowed to provide written feedback about the service, for example, by filling out a questionnaire or using an idea box.	1	79	91	69	80
*Stage: Continued support*						
1.36	Services should also provide support for parents of YP, for example, family therapy, parent evenings.	2^c^	81	86	64	83
1.37	Healthcare providers should attempt to reach YP when they fail to show up without giving notice.	1	78	89	68	74
1.38	If YP have multiple healthcare providers, services should make sure one of the healthcare providers takes up the role of coordinator.	2^c^	70	76	46	59
1.39	Services that offer help to YP must set up a professional network that the young person can count on, for example, buddies, counsellors, coaches and mental health professionals.	2	82	70	/	/
1.40	Services should gradually decrease the intensity of the provided care, for example, by having a short trial period in which YP are expected to be more autonomous.	1	98	86	74	66
1.41	Services should organize a meeting with the entire network of the young person (informal and professional) before ending the treatment.	2^c^	89	97	78	86
1.42	Services should facilitate that YP can re‐engage with the service if necessary.	1	93	85	58	46
1.43	Services should allow YP to stay in touch with the service after the end of their trajectory, for example, by telephone or dropping in for activities.	1	81	97	58	74
1.44	Services should allow professionals to provide blended care.	3^c^	73	86	71	67
1.45	Services that offer help to YP should not just address mental health but also make sure YP get help for other life domains, for example, finding work, education, housing and a place in society.	1	88	100	/	/
1.46	Finding meaningful day activities should be an important focus in the treatment of YP.	1	85	97	/	/
1.47	Services should stimulate YP that have experience with the service to become a buddy to YP that have just gotten into the service.	3^c^	84	83	74	57

Abbreviation: YP, young people.

^a^ Feasibility was not rated for items 1.8, 1.17–1.25, 1.39, 1.45 and 1.46.

^b^ Item reformulated after round 1.

^c^ New item after round 1.

**Table 3:** Items with consensus on importance (70% or more endorsing a score of 7–9) in one panel only.

Number	Description of item upon consensus	Round	% Agreement importance	
			Public	Professional
*Stage: Initial contact*				
	Consensus in the public panel only			
2.1	Services should inform school‐age YP by speaking at schools and organizing class visits.	1	76	64
2.2	Services should offer sufficient privacy in the entrance or waiting area, both to keep people from looking in as well as between people inside.	2^a^	76	64
	Consensus in the professional panel only			
2.3	Services should make their website attractive to YP, for example, by including pictures and giving straightforward explanations.	1	69	81
2.4	Services should open their doors so that YP and parents can get to know them, for example, by organizing open days and visits.	2	69	73
2.5	Services should allow YP to make and place decorations, for example, on the walls.	1	59	94
*Stage: Treatment*				
	Consensus in the public panel only			
2.6	Services should enable YP to read everything what is written about them and their trajectory, for example, in a medical record.	2	81	62
2.7	Services should have YP with lived experience in the team of healthcare providers.	1	76	68
2.8	Services should be located in a green environment or create a green area, for example, a garden.	3^b^	94	66
2.9	*For inpatient treatment*: Services should designate a specific place where YP can receive a visit from their pet(s).	2^b^	84	68
	Consensus in the professional panel only			
2.10	Getting a diagnosis from healthcare providers can be helpful for some YP.	3^b^	68	86
2.11	Services should allow YP to smoke outside but without providing extra amenities.	3^b^	68	83
2.12	Services should invite YP to join the professionals' meetings when their trajectory is being discussed.	1	66	91
2.13	YP must be allowed to give verbal feedback about the service, for example, by joining a meeting with management or setting up a youth council.	1	67	75
2.14	YP should be asked their opinion on the floor plan of a new service, for example, which room do they want as quiet room or recreation room.	3^b^	60	79
*Stage: Continued support*				
	Consensus in the public panel only			
2.15	Services should only end treatment when YP feel ready.	1	88	54
2.16	Services should enable healthcare providers to stay in contact with YP through digital communication, for example, to follow up on how the YP are doing or to remind them of an upcoming appointment.	1	73	69
	Consensus in the professional panel only			
2.17	Services that offer help to YP should encourage YP to strengthen (and sometimes restore) ties with parents, friends and so on, in addition to developing new relationships.	1	60	86
2.18	Services should involve support figures of YP, for example, parent(s), sibling(s) and friend(s), for example, by organizing joint consultations.	1	68	94
2.19	Services should maintain good contacts with leisure, for example, gym, music school and volunteer organizations, for example, an animal shelter in the area.	2^b^	68	76
2.20	Services that offer help to YP should facilitate meaningful interaction between YP so they can share their stories.	1	64	81

Abbreviation: YP, young people.

^a^ New item after round 1.

^b^ Item reformulated after round 1.

Consensus across both panels was reached for 67 items (93%), leaving five items (7%) that were deemed unimportant (see Table 3 in the Supporting Information Appendices).

We structured the items according to three stages (‘initial contact’, ‘treatment’ and ‘continued support’) along the care pathway (see Tables 2 and 3).

#### 3.3.2 (Dis)agreement on perceived feasibility

Within both panels, levels of agreement for feasibility were much lower (see Table 2). Only the items for which consensus on importance within both panels was reached, were considered.

Only nine out of the 47 consensus items (19%) were deemed to be highly feasible for clinical practice (a score of 7 or above on a 9‐point Likert scale as endorsed by ≥70% of the panelists) by both panels. This included the provision of an information brochure (1.3), a referral being unnecessary unless the service is highly specialized (1.6) and inviting the young person before the start of treatment (1.9). Offering free WIFI (1.13), creating a pleasant entrance/waiting area (1.14), having a cozy communal area (1.16) and a quiet room (1.26) were not only considered highly relevant to the youth‐friendliness of a service but also highly feasible. Furthermore, both panels thought working with a care plan (1.31) and organizing a network meeting before the end of treatment (1.41) were easy to implement.

Finally, eight items (1.2, 1.5, 1.10, 1.12, 1.35, 1.36, 1.37 and 1.43) were considered highly feasible by the professional panel only, while five items (1.15, 1.30, 1.40, 1.44 and 1.47) were deemed to be highly feasible by the public panel only.

### 3.4 Qualitative results

**Figure 2:**
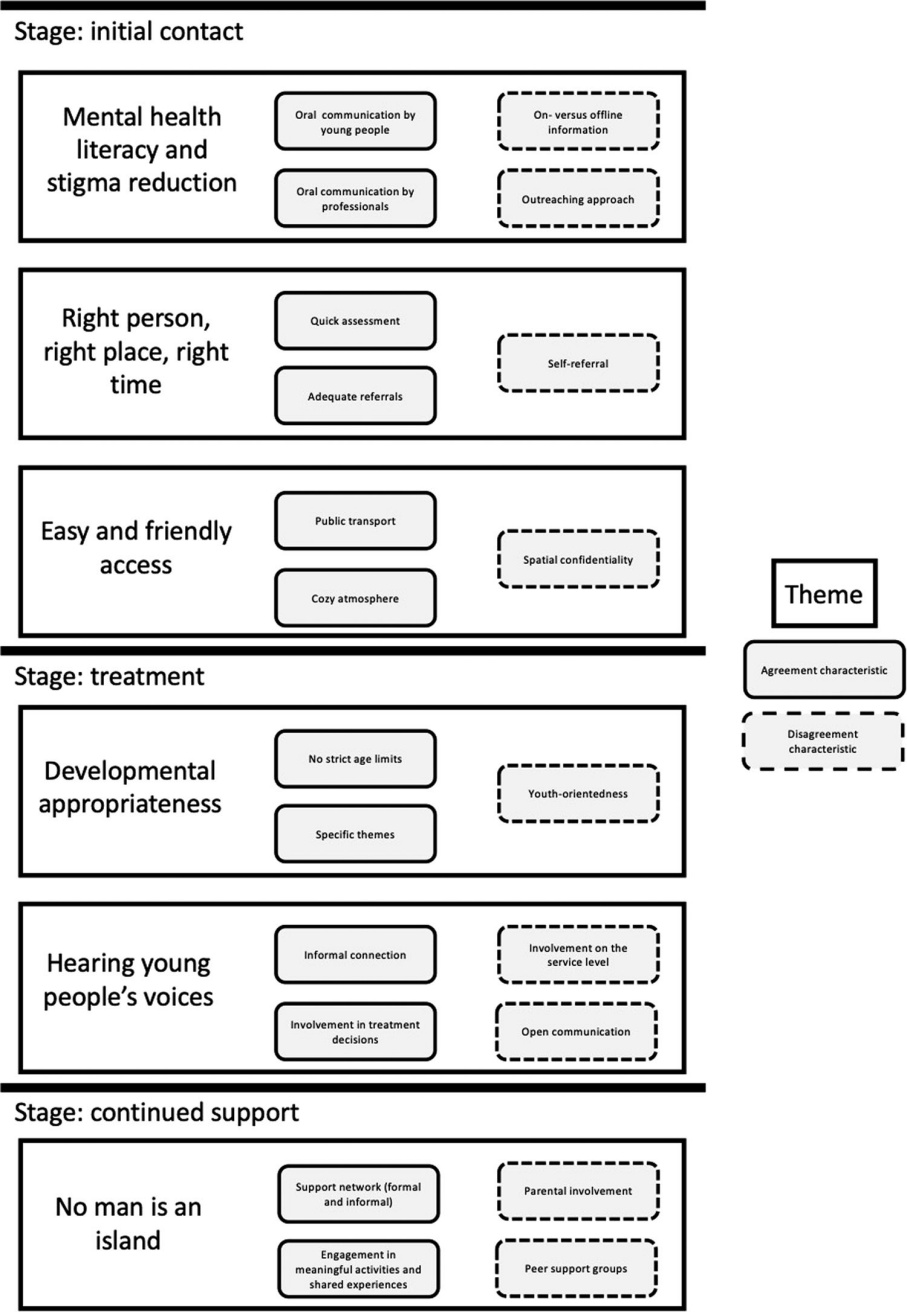
Overarching themes identified by thematic analysis with (dis)agreed upon characteristics across three stages.

Our thematic analysis resulted in the identification of six themes that we structured according to the three aforementioned stages (‘initial contact’, ‘treatment’ and ‘continued support’) (see Figure 2). For each theme, we provide an exemplary quote and discuss the characteristics for which there was (dis)agreement within and across both panels. Additional exemplary quotes can be found in Table 4 in the Supporting Information Appendices.

#### 3.4.1 Initial contact

The first theme we identified based on the thematic analysis was ‘*mental health literacy and stigma reduction*’.

I was sent to a service about which I could not find much information online. This made me anxious the first time I was there. (Young person, round 2, item 1.3)

Especially young adults raised concerns about the outdated image some people, mostly from older generations, have of how mental health services operate. Emphasis was put on (1) improvement of knowledge about which services exist, and (2) giving a realistic image of how these services operate. The latter was considered important to diminish stress and anxiety during the help‐seeking process. Both panels agreed that multiple sources of information would be most efficient with a preference for information that is conveyed orally, preferably by young adults with experience. Online and visual media were a good source of information for most young adults, although some found more traditional measures (e.g., an information brochure) more suitable due to concerns about privacy and reliability. Another possibility lies in the face‐to‐face interaction with professionals either in places that young adults visit (i.e. the ‘outreaching’ approach) or in the service itself. While the public panel shared a strong preference for an outreaching approach, the professional panel considered organized visits or open days more realistic.

The second theme was ‘*right person, right place, right time*’.

Looking for help is hellish for parents. The many waiting lists and not knowing if you meet the criteria makes it miserable. Waiting a long time for a ‘no’ is totally unnecessary. (Parent, round 2, item 1.7)

Having to wait for a response from a service whether the young person can get help or not was experienced by young adults as stressful and even harmful when the situation deteriorated in the meantime. Therefore, the panels agreed that services should perform a quick and adequate assessment, referring to another service if necessary, to avoid dropout. Up‐to‐date information on available services, for instance, on a website, and collaborations between services in an established network would make referrals less complicated. Both panels agreed that CAMHS and AMHS professionals should assist young adults during the help‐seeking process to find the most suitable service, given the complexity of the landscape.

Many experts from the public panel and professional panel thought that this assessment should be based on a conversation with the young person to clarify the individual's needs, expectations and motivation following self‐referral. Others thought it advisable to receive information from an involved healthcare professional, for instance, by use of a referral letter.

Another important theme in this stage was ‘*easy and friendly access*’.

That's the first impression you get of an organization [waiting area/entrance]. If that place is not inviting, people could drop out before they even start. (Young person, round 2, item 1.14)

Both panels agreed that services located close to public transport are more accessible to young adults because they can visit them independently. Furthermore, being welcomed by a person upon entering can have a comforting effect on young adults who are feeling anxious when consulting a service for the first time. A cozy atmosphere rather than a hospital‐like environment puts young adults at ease. Most young adults opposed, however, the colocation of services in places they often visit, which contrasts with the view of professionals that this would increase the visibility of services. The most frequently mentioned explanation was that the likelihood of being seen by peers increases. Also, within the walls of the service, the public panel demanded attention to their privacy. Suggestions to increase spatial confidentiality included windows with frosted glass, a designated waiting area separate from the entrance and the creation of nooks. Professionals, on the other hand, were worried that these measures would reinforce the mental illness stigma.

#### 3.4.2 Treatment

The first theme that emerged from the thematic analysis for this stage was ‘*developmental appropriateness*’ of treatment.

—a service focuses on either children and adolescents or adults. Adults, however, are such a broad target group and the same approach does not apply to a 20‐year‐old than a 60‐year‐old. (Professional, round 2, item 1.17)

Both panels agreed that the adoption of strict age limits in CAMHS and AMHS was not helpful given that this might lead to discontinuity of care. Rather than looking at the age of the young person, it was suggested to focus on the individual's maturity. Adopting a developmentally appropriate approach translates to treatment as well, in the sense that it should be focused on highly relevant themes to young adults' reality, such as developing a positive self‐image and identity formation.

Within both panels, there was substantial disagreement about how this theme should be implemented in clinical practice. One group of participants was more youth‐oriented: they were strong advocates for services dedicated to youth, in which healthcare professionals from CAMHS and AMHS partnered. It was noted that young adults would feel more comfortable with individuals their age who had not yet adopted an ‘illness identity’. This group also opposed diagnostic categorization to avoid ‘labelling’. Other participants did not mind a mixed group, stating that all age groups could learn from each other. They also looked more favourable upon receiving a diagnosis for reasons of recognition and the possibility of meeting like‐minded persons.

A second important theme was ‘*hearing young people's voices*’.

It depends: some young people like their previous healthcare provider to coordinate [with their current healthcare provider] so they don't need to retell their story. This can also help the healthcare provider gain some insight and build a relationship with the young person. This simply must be communicated well, and the young person should be consulted. Ask the young person whether this has an added value or not. Every person has different needs and preferences. (Young person, round 1, item 1.29)

Both panels agreed that performing a relaxing activity to informally connect with a healthcare professional might make it easier for young adults to talk about difficult topics. Examples included taking a walk outside together or playing a game in a recreation area while engaging in informal conversation. Young adults also put a lot of emphasis on being allowed to make their own choices for their treatment. The professional panel agreed that providing young adults with correct information is important so they can make well‐informed decisions. Moreover, young adults appreciated being asked for feedback on how the service operates. Within the public panel, there was disagreement, however, on how young adults should be involved on the service level. Some young adults only felt comfortable filling in a questionnaire anonymously, while others would appreciate to convey their opinions orally in a meeting with healthcare professionals.

Finally, open communication was deemed important to avoid distrust. However, there was no agreement between and within the panels on how this should be put into practice. Professionals thought, for instance, that it would be interesting to invite the young person to a team meeting in which the progression of the young person is discussed, whereas most participants in the public panel found this too daunting. The public panel on the other hand was more interested in being able to read the young person's files and reports, which professionals were less enthusiastic about because they felt this would hamper their ways of communicating.

#### 3.4.3 Continued support

The last theme we identified based on the thematic analysis was ‘*no man is an island*’.

Young people can isolate themselves from other people subconsciously because of their problems. Then the other party doesn't know what to do anymore and drops the young person. I find it important to restore relationships, no matter how hard, it will always be your child, brother, family, friend, etc… (Parent, round 1, item 2.17)

All experts agreed that having a strong, informal support network is essential. A lot of young adults felt that their parents still played an important role in their lives. Both panels identified the need for structural support for the family, for instance, family therapy or group therapy for parents. However, parental involvement in treatment was not self‐evident. Some young adults were concerned that restoring existing connections might not have been beneficial in their case and stressed that young adults should be asked for permission first. In case a young person does not want their family involved, the public panel suggested that the participation of (a) friend(s) or confidant would be a good alternative.

The panels also agreed that young adults should feel like they can rely on their formal network (i.e., their healthcare professional(s)), especially when it comes to the end of their care, which should be a gradual, carefully prepared process. Some participants in the public panel and professional panel were keen to use digital communication such as text messages outside consultation hours to ensure continuity of care, especially when the intensity of care is decreased. Some professionals, however, were insecure about using these media, worrying about technological difficulties, professional boundaries, liability and privacy.

Additionally, feeling useful and productive, feeling that you are part of something and that you contribute to society are important protective elements according to both panels. Therefore, both panels thought that life domains such as studies, work, housing, relationships/sexuality and so on should not be neglected and young adults should be stimulated (and challenged) to partake in activities outside mental health care. Young adults appreciated it when the service explored the options with them and alleviated their anxiety, by going with them the first time for instance.

Finally, both panels agreed that being able to share experiences with peers could have a positive impact on young adults. Perceived benefits were feeling truly understood, learning from each other (e.g., getting useful tips) and being less lonely. The panels agreed on a buddy system, which entails that young adults who have longer experience with the service help the new young adults feel comfortable and answer their questions. Participating in peer support groups was not considered helpful by all young adults, however: concerns were potentially being triggered by hearing about similar difficulties and a strong focus on problems or problematic behaviour.

## 4 DISCUSSION

### 4.1 Agreement on youth‐friendly service characteristics

For 65% of the items in the Delphi questionnaire, consensus on importance was reached within both panels. This confirms that the characteristics identified by Hawke et al.^17^ are highly relevant when it comes to applying the concept of youth‐friendliness to mental health service development, design and delivery.

In terms of the initial contact stage, we can conclude that there is still a long way to go to make services more visible, appealing and readily available for young adults. Battling stigma is an important social responsibility. However, even if this is overcome, all stakeholder groups mentioned that finding and getting adequate care is a complex, confusing and often lengthy process, which was also found in other studies.^8^, ^13^, ^33^ One promising approach is involving youth peer support workers (YPSWs) to assist in the help‐seeking process.^34^ Young adults in this study indeed found it beneficial that a YPSW would take up a navigating role. They did not all feel comfortable, however, to share their story with them and depend on them for emotional support, mostly due to previous negative experiences. Future efforts should be geared towards sufficient financing, supervision, training of the YPSWs and the nonpeer staff, to successfully implement a peer support model.^35^, ^36^

The items that reached consensus pertaining to the treatment stage indicated that although not all youth‐friendly characteristics, such as involvement in treatment decisions, are youth‐specific, certain themes (e.g., identity formation, self‐management, emotion regulation, narratives of hope) seem to particularly resonate with the young adult population. In recent years, new youth‐focused mental health teams have adopted a more transdiagnostic and person‐centred approach to treatment.^37^, ^38^, ^39^ An important factor that can affect person‐centred care is the capacity of young people to be involved during times of crisis.^40^ Previous studies have shown, however, that professionals should be careful not to underestimate their capacity or willingness to participate.

Our study also revealed that all stakeholder groups thought the establishment of continued support to be an important outcome of mental health treatment. Not only having a strong support network was considered beneficial but also engaging in meaningful activities and sharing experiences with other young adults. Our study thus shows parallels to the recovery model that represents a shift from a focus on illness to strengths, deficit to resilience and individual problems to context as the most effective path to mental health care.^41^

Finally, it is important to note that although there was substantial between‐ and within‐panel agreement on importance, the agreement scores for perceived feasibility in clinical practice were much lower. Professionals considered more items to be highly feasible than young adults and parents. A possible explanation might be that the professionals who participated in the study were more likely to have adopted a youth‐friendly approach in their practice already, whereas this was experienced less by young adults and parents in the services they had consulted.

### 4.2 Disagreement on youth‐friendly service characteristics between and within the panels

One of the recommendations made in earlier publications to facilitate initial contact with services is integrating them, making them ‘one‐stop shops’, ideally by colocation of mental health services with other care provisions for youth.^19^ However, in our study especially young adults strongly opposed colocation of services in proximity to places they often visit due to privacy concerns. A possible explanation for this is the predominance of attitudinal barriers to help‐seeking, among which is embarrassment, over structural barriers.^8^, ^42^ Also, inside services young adults and parents appreciated privacy‐enhancing measures. A Swedish study about youth‐friendly health services also found that placing services in well‐known locations might hinder access and spatial confidentiality should be warranted (e.g., isolated and soundproof rooms, keeping doors closed).^43^ At the same time, though, services need to project an image of openness, which was also supported by the professionals in our study who worried about reinforcing the mental health taboo. Rather than involving young adults to create a cozy space with youthful decorations, it might be interesting to ask their input on these matters.

Furthermore, although young people are avid users of social media and are very likely to pose mental health queries online,^44^ our study confirmed concerns with privacy and credibility of the information.^45^ Mental health professionals/services do well to consider whether they want to deliver broad‐casted (i.e., one‐to‐many) or narrow‐casted (i.e., information for a specific audience) content.^46^ In case of the latter, more traditional measures, such as an information brochure, might be more suitable.

Concerning the items covering the treatment stage, there is clear between‐ and within‐panel disagreement on involvement of young adults on the service level. One study showed that young adults who wanted to share their ideas and opinions about service design and delivery were passionate to help overcome barriers to help‐seeking and wanted to build confidence, social skills and make new friends.^47^ It is clear from our study, however, that not all young adults feel ready to be involved and that involvement should not be limited to one particular format, such as an advisory board, but should be attempted in multiple ways. Important conditions for optimal involvement are openness from the professionals' perspective, clarity of roles, skills training and the inclusion of young adults of different backgrounds and with different experiences.^48^

In terms of the continued support stage, parental involvement was a particularly sensitive topic for young adults in the public panel. At the beginning, but also during and at the end of treatment, healthcare professionals should check with young adults whether they are open to this and how this can be realized. During young adulthood, personal independence is gradually gained and often not complete.^49^ While connectedness and ‘warm’ involvement of parents without them being overly intrusive and controlling are associated with positive development during this life stage,^50^, ^51^ trust‐related ruptures in the caregiver–child relationship have a negative impact on young adults' ability to explore the environment and result in less adequate emotional regulation skills and identity formation challenges.^52^ From the parents' perspective, involvement is often preferred, even after their child is legally considered an adult and support is welcomed.^53^, ^54^, ^55^ An inpatient unit for young adults in Belgium adapted attachment‐based family therapy to their setting and found that treatment involvement was increased, the number of episodes of seclusion decreased and relationships with primary caregivers were more collaborative.^52^ It is important to note, however, that in most European countries the key role of the family and parent management techniques are covered in child and adolescent psychiatry training, while this is much less the case in adult psychiatry.^56^

### 4.3 Limitations

There are limitations to this study that warrant consideration.

First, due to recruitment of young adults via mental health services and professionals, the sample was characterized by an over‐representation of female participants. Additionally, although we targeted young people between the ages of 16 and 27, no minors participated in the study. Also, only 3 young people were born outside Belgium. Young adults who felt more strongly about the topic might also have been more inclined to partake.

Second, it is important to note that the number of parents in the public panel was very small and that they were all mothers. Nevertheless, the panels still consisted of over 20 members, which is relevant in terms of stability.^24^

Third, the fact that consensus on importance was reached for many items begs the question whether there was sufficient room for disagreement. We confronted the panelists, however, only in the third round with the median scores of the whole sample and our results show that within‐panel agreement was larger than between‐panel agreement.

Finally, although the attrition rate for the online questionnaire rounds was quite low,^57^ there was a low participation rate in the physical meeting. Therefore, it is noteworthy that in the physical meeting consensus was not reassessed, but the goal was rather to get a better understanding of the discrepancies between both panels.

### 4.4 Implications for future research and clinical practice

We recommend that the 47 items for which consensus was reached within both panels should be used as a checklist in terms of youth mental health service development, design and delivery. Services with limited resources can prioritize based on the feasibility scores that we obtained in the study. Further research should be focused on mapping out the youth‐friendliness of existing mental health services for young adults considering young adults', parents' and professionals' perspectives. Young people that attempt to seek help for the first time should also be consulted to evaluate whether the initial contact items adequately capture their preferences for the youth‐friendliness of a service. Given the low feasibility scores in the study, a qualitative investigation of implementation barriers could provide more insight.

The characteristics for which there was disagreement between and within the panels should inspire an ongoing dialogue between young adults and professionals with an emphasis on *with* young adults instead of *for* young adults. It is clear that when it comes to these characteristics, there is no one‐size‐fits‐all and that services should provide multiple possibilities. This demands more flexibility from a mental health care system that is currently dichotomized and does not allow for extensive individualization. Finally, our study has clearly shown that parents can be important allies and should be part of discussions on the individual and service level. This means that professionals working with young adults should feel comfortable operating within a trialogue. Efforts should be dedicated towards implementation of cross‐training in which CAMHS and AMHS clinicians receive training together on youth‐specific topics.

## 5 CONCLUSION

This Delphi study investigated to what extent young adults, parents and professionals (dis)agreed about the relevance of implementations of previously identified characteristics to the youth‐friendliness of services. For many items consensus was reached within the public panel and the professional panel. These items can be used by services as a checklist for youth‐friendliness. For implementation, it is important to note that participants showed more insecurity about the feasibility of these items in clinical practice. Finally, our study also revealed some topics of discussion that invite a continuous trialogue between young adults, parents and professionals in terms of treatment and service design and delivery.

## AUTHOR CONTRIBUTIONS

Eline Wittevrongel, Ruud van Winkel, Dieter Baeyens and Marina Danckaerts developed the concept for the study and designed the study. Eline Wittevrongel and Ruud van Winkel coordinated the study. Eline Wittevrongel, Melina Versyck and Eline Camp collected the data. Eline Wittevrongel, Melina Versyck, Eline Camp and Maarten Jackers performed the analyses and interpreted the data. Eline Wittevrongel and Maarten Jackers drafted the manuscript. Ruud van Winkel, Laura Colman, Geert Everaert, May Vrijens, Dieter Baeyens and Marina Danckaerts provided critical revisions of the manuscript. All authors read and approved the final manuscript.

## CONFLICT OF INTEREST STATEMENT

The authors declare no conflict of interest.

## ETHICS STATEMENT

The study received ethical approval from the UZ/KU Leuven Medical Ethics Committee (S64938) and was performed according to the principles of the Declaration of Helsinki. Electronic informed consent was obtained from all participants in this study.

## Supporting information

Supporting information.

## Data Availability

The data that support the findings of this study are available on request from the corresponding author. The data are not publicly available due to privacy or ethical restrictions.
