# Supplementary material for: How to make mental health services more youth‐friendly? A Delphi study involving young adults, parents and professionals
Source: Health Expect. 2023 Aug 22;26(6):2532–48. doi: 10.1111/hex.13832 (PMC10632649; doi:10.1111/hex.13832)
Supplement: Supplementary file 1 — Supporting information. [file HEX-26--s001.docx]

**Appendices**

*Table 1: Overview of youth-friendly service characteristics identified by Hawke et al.^1^ and their correspondence with the Delphi items*

| **Category identified by Hawke et al.** | **Characteristics identified by Hawke et al.** | **Items in Delphi study*** |
| --- | --- | --- |
| Organization and policy characteristics | Youth voice: youth engagement at the organization/policy level | 1.35, 2.13 |
|  | One-stop shop/integrated services | 1.8, 1.45, *N2*** |
|  | Confidentiality/privacy | 2.2† |
|  | Appropriate promotional approaches | 1.1, 1.4, 2.1, 2.4, *N1*** |
|  | Technological platforms | 1.2, 2.3, *D1**** |
|  | ‘Safe’ space versus ‘brave’ space | 1.25 |
|  | Transitional age focus | 1.10†, 1.17-1.24, 1.46 |
|  | Inclusive and culturally diverse services | 1.28 |
|  | Terminology | 2.10 |
| Environment characteristics | Youth voice: youth engagement in environment development | 2.5, 2.14 |
|  | Physical layout and décor | 1.13, 1.14†, 1.26, 2.11 |
|  | Informational materials | 1.3 |
| Staff and service provider characteristics | Paradigms of working with youth | 1.37 |
|  | Youth voice: youth as service providers | 1.47† |
|  | Young staff members | 2.7 |
|  | Welcoming staff | 1.12†, 1.27 |
|  | Service provider values & attitudes | 1.29, *N4*** |
|  | Communication and counseling skills | 1.38† |
| Treatment/service characteristics | Location and access | 1.11, *N3*** |
|  | Appointment and wait times | 1.32†, 2.16 |
|  | Cost‡ | / |
|  | Youth-practitioner fit | 1.33 |
|  | First contact and assessment | 1.5, 1.6, 1.7† |
|  | Youth voice: youth involvement in treatment decisions | 1.9, 1.30, 1.31, 1.34, 2.6, 2.15 |
|  | Individualized response | 2.12, 1.39, 1.40, 1.41†, 1.42, 1.43 |
|  | Recreational approaches | 1.15, 1.16, 2.8, 2.19, *D2**** |
|  | Group therapy and group activities | 2.20, *N5*** |
|  | Artistic and innovative approaches | 2.9, 1.44† |
|  | Caregiver involvement | 1.36†, 2.17, 2.18 |

^1^Adapted from: Hawke LD, Mehra K, Settipani C, et al. What makes mental health and substance use services youth friendly? A scoping review of literature. *BMC Health Serv Res.* 2019;19(1):257. doi:10.1186/s12913-019-4066-5

*Number of items corresponds with numbers in Table 2 and 3 in manuscript

**Items for which consensus was not reached, for description of items see Table 3 in Appendices

***Items deleted after first round due to overlap with other item, for description of items see Table 2 in Appendices

†Items added after the first round

‡No items were added to the questionnaire relating to the service attribute cost because a preference for low cost is self-evident.

*Table 2: Overview of descriptions of reformulated and deleted items in Delphi questionnaire*

| **Number*** | **Description of characteristic in the first round** | **Description of characteristic upon consensus** |
| --- | --- | --- |
| 1.1 | Services should use influencers to inform YP about how they operate. | Community mental health services should fight stigma by collaborating with influencers. |
| 1.2 | Services should have their own Facebook page. | Community mental health services should make use of social media to gain more exposure. |
| D1 | Services should have their own Instagram profile. | *Incorporated in item 1.2.* |
| 1.3 | Services should hang up posters and distribute flyers in public spaces to promote themselves. | Services should have a folder or information brochure to inform YP and parents about how they operate. |
| 1.6 | Services should not ask for referrals e.g. from the general practitioner of YP. | Services should only ask for referrals if they are highly specialized, thus only offering help to a specific group. |
| 1.8 | Services should have a ‘no wrong door’ policy, meaning that every young person with any kind of complaint or problem is welcome in the service. | YP that don’t know which service to get help from should be assisted by professionals in community services that direct them to ‘the right door’. |
| 1.13 | Services should create a comfortable waiting area with drinks, food and free WIFI. | Services should have free WIFI. |
| 1.17 | Services should provide care specifically to YP: YP should not be mixed with adolescents and (older) adults. | Services that offer help to YP should adapt their provision of care to the specific needs of YP. |
| 1.24 | Challenging YP to push their boundaries should be an important focus in the treatment of YP. | Discovering your own limits (What is my comfort-, stretch- and panic zone?) should be an important focus in the treatment of YP. |
| 1.33 | Services should allow YP to choose their healthcare provider within the service. | When YP and/or their parents don’t feel at ease with the healthcare provider, YP should be given the opportunity to switch providers within the service. |
| 2.8 | Services should be located in a green environment. | Services should be located in a green environment or create a green area e.g. a garden. |
| D2 | Services should have a garden e.g. to take a walk in or grow fruits and vegetables. | *Incorporated in item 2.8.* |
| 2.9 | Services should have a space for animals e.g. cats, a horse. | *For inpatient treatment:* Services should designate a specific place where YP can receive a visit from their pet(s). |
| 2.10 | Services should perform diagnostic tests to gain insight into YP’s difficulties and start treatment based on the results. | Getting a diagnosis from healthcare providers can be helpful for some YP. |
| 2.11 | Services should have a designated area where YP are allowed to smoke. | Services should allow YP to smoke outside but without providing extra amenities. |
| 2.14 | Services should create a floor plan with the help of YP e.g. YP get to decide which room they want as quiet room or recreation room and which sport amenities there will be. | YP should be asked their opinion on the floor plan of a new service e.g. which room do they want as quiet room or recreation room. |
| 2.19 | Services should have a sport center or sport amenities e.g. a trail for walking, an exercise room or a skatepark. | Services should maintain good contacts with leisure e.g. gym, music school and volunteer organizations e.g. an animal shelter in the area. |

Abbreviation: YP = young people.

*Number of items corresponds with numbers in Table 2 and 3 in manuscript

*Table 3: Overview of % agreement on importance (% participants endorsing a score of 7-9) across both panels*

| **Number*** | **% Agreement** |
| --- | --- |
| 1.1 | 73 |
| 1.2 | 80 |
| 1.3 | 76 |
| 1.4 | 81 |
| 1.5 | 87 |
| 1.6 | 72 |
| 1.7 | 100 |
| 1.8 | 97 |
| 1.9 | 93 |
| 1.10 | 94 |
| 1.11 | 95 |
| 1.12 | 85 |
| 1.13 | 83 |
| 1.14 | 89 |
| 1.15 | 78 |
| 1.16 | 88 |
| 2.1 | 71 |
| 2.2 | 71 |
| 2.3 | 74 |
| 2.4 | 71 |
| 2.5 | 75 |
| 1.17 | 93 |
| 1.18 | 95 |
| 1.19 | 100 |
| 1.20 | 88 |
| 1.21 | 97 |
| 1.22 | 92 |
| 1.23 | 88 |
| 1.24 | 97 |
| 1.25 | 95 |
| 1.26 | 90 |
| 1.27 | 85 |
| 1.28 | 78 |
| 1.29 | 76 |
| 1.30 | 87 |
| 1.31 | 95 |
| 1.32 | 80 |
| 1.33 | 73 |
| 1.34 | 80 |
| 1.35 | 84 |
| 2.6 | 73 |
| 2.7 | 72 |
| 2.8 | 80 |
| 2.9 | 77 |
| 2.10 | 77 |
| 2.11 | 75 |
| 2.12 | 78 |
| 2.13 | 71 |
| 2.14 | 70 |
| 1.36 | 83 |
| 1.37 | 83 |
| 1.38 | 73 |
| 1.39 | 77 |
| 1.40 | 92 |
| 1.41 | 92 |
| 1.42 | 89 |
| 1.43 | 88 |
| 1.44 | 79 |
| 1.45 | 94 |
| 1.46 | 91 |
| 1.47 | 83 |
| 2.15 | 72 |
| 2.16 | 71 |
| 2.17 | 72 |
| 2.18 | 80 |
| 2.19 | 71 |
| 2.20 | 72 |
| N1 Services should make an informational video about how they operate. | 20 |
| N2 Services should be co-localized with other services for youth e.g. housing services, financial aid. | 14 |
| N3 Services should be located in close proximity to places that young people visit regularly e.g. schools/campus, youth cafés. | 18 |
| N4 Services should consult YP before hiring new employees to find out which characteristics they find important in a mental health professional. | 46 |
| N5 Services should bring YP into contact with other YP who used to get help from the service after treatment e.g. by means of a moderated Facebook group. | 35 |

Abbreviation: YP = young people.

*Number of items corresponds with numbers in Table 2 and 3 in manuscript

*Table 4: Exemplary quotes corresponding to the themes identified by thematic analysis*

| **Theme** | **Agreement/disagreement** | **Exemplary quotes** |
| --- | --- | --- |
| Mental health literacy and stigma reduction | Agreement | *“—being admitted to inpatient care is often still taboo because people have an unrealistic image of psychiatry. For me personally, this has caused a substantial delay in treatment…it took a long time for my mother to agree to my admission.”*  (Young person, round 2, item 2.4)  *“For some young people it is helpful to see well-known people they look up to also dealing with things. It does not need to be taboo; you shouldn’t feel ashamed of having psychological difficulties.”*  (Young person, round 2, item 1.1)  *“For my daughter it is necessary that she can picture where she will end up, so she doesn’t have to ask questions because she knows ‘her way’ already or recognizes things/people.”*  (Parent, round 2, item 2.4)  *“There are young people that are willing to help others by drawing on their own experiences, which makes it easier for someone else to talk about it. They want to tell their story and motivate others to take the step towards help. They are experts by experience…”*  (Parent, round 2, item 1.4)  *“I think young adults often look things up online before making contact. However, I think that face-to-face contact to get explanations and to be able to ask questions is also very important.”*  (Professional, round 1, item 2.3) |
|  | Disagreement | *“I think that a folder or brochure can certainly be useful. Especially because young people (and parents) can go through the information at their own pace. It also becomes more tangible than looking up information on a website for instance.”*  (Young person, round 2, item 1.3)  *“Do not organize visits or open days when patients are present. Then you feel like you’re an animal in a zoo.”*  (Young person, round 1, item 2.4)  *“It* [presence of services on social media] *can be a good measure for young people that don’t have the courage to go looking for mental health services themselves.”*  (Young person, round 2, item 1.2)  *“Most young people use social media, so this is the ideal way to reach them.”*  (Young person, round 2, item 1.2) |
| Right person, right place, right time | Agreement | *“Just a little message that says ‘we have received your request’; ‘we are working on it’ can be very calming.”*  (Young person, round 2, item 1.7)  *“I have often been on a waiting list for months to be told after a first intake interview that I could better go elsewhere, only to end up on the next waiting list.”*  (Young person, round 2, item 1.7)  *“It is important that services know the landscape to meet the needs of young people, to be able to inform them properly and refer them correctly. Knowing each other often also inspires trust and hope in the young person who asks for help or has to turn to someone. It makes for a more reliable and comprehensible network.”*  (Professional, round 2, item 1.10) |
|  | Disagreement | “[Young people contacting the service themselves] *shows their motivation. Unfortunately it happens quite a lot that other professionals or parents put in a request for help without the young person knowing or wishing it.”*  (Professional, round 1, item 1.5)  “[Young people contacting the service themselves is] *the best way to start treatment. It shows initiative and you get to know the young person from the start.”*  (Professional, round 1, item 1.5)  *“Why should a doctor* [general practitioner] *be allowed to decide how bad someone feels in a particular situation. That doctor sees that young person for only 10 minutes and sees maybe 50 people a day. It’s impossible to say whether the problems are severe enough for further treatment or not.”*  (Young person, round 1, item 1.6)  *“*[Needing a referral letter is important] *to allow for stepped care.* *First you go see a psychologist (there you won’t need a referral letter in most cases) and if necessary other steps can be taken after.”*  (Young person, round 3, item 1.6) |
| Easy and friendly access | Agreement | *“It* [the service being close to public transport] *is an important benefit to me, for the journey but also because it allows independence (doing it on your own without a parent).”*  (Young person, round 1, item 1.11)  *“Do not put waiting chairs in a passageway that is too narrow to be able to remain seated when professionals are constantly walking by. Do not put up a loud ticking clock that is hard to ignore.”*  (Parent, round 2, item 1.14)  *“It shouldn’t look like a psychiatric facility or hospital.”*  (Young person, round 2, item 1.14)  *“I think a green area is very important but it doesn’t have to be a vast domain. A box with spices or flowerpot can be sufficient. I think that greenery can give a sense of freedom and homeliness in a professional environment that often is sterile.”*  (Young person, round 3, item 2.8) |
|  | Disagreement | *“Co-location lowers the threshold because you’re already there, but also increases the likelihood of being seen by peers. It is less anonymous which can be important.”*  (Young person, round 2, item N2)  *“It is less strange for young people because they already know the place but others can see you entering the service. I also think you would feel less comfortable because you might run into people you know. Many young people do not want certain friends to know…”*  (Young person, round 2, item N2)  *“As a parent and as a young person, the first step* [towards getting help] *is often a difficult one and you think you are the only person dealing with these problems. A little privacy in the beginning is therefore definitely recommended, also to put you a little more at ease...”*  (Parent, round 2, item 2.2)  *“For me there is a difference between sitting in the hall right behind the entrance or sitting in a designated waiting area…At the entrance everyone just comes in: the mail man, the supplier that comes to drop of something,…People that have nothing to do with the actual service.”*  (Parent, focus group, item 2.2) |
| Developmental appropriateness | Agreement | *“—Soon you will be 18 years old…We can’t refer you to child and adolescent mental health services; they say they won’t get to it anymore. On the other hand, we can’t make a referral to adult services because you’re not 18 yet…There is a large gap.”*  (Professional, focus group, item 2.15)  *“Age is a number that doesn’t align with mental age or maturity. Age shouldn’t be a factor. Young people should be in a group they feel most comfortable with.”*  (Parent, round 1, item 1.17)  *“Young people often do not know what their strengths are. It is good to work on this. Especially if you look upon yourself in a negative way all the time.”*  (Young person, round 1, item 1.18)  *“I don’t know who I am, I’m trying to discover this so I find this theme* [identity formation] *important. I don’t think I’m the only one struggling with this.”*  (Young person, round 1, item 1.19)  *“Almost all young people have trouble regulating their emotions which is typical for adolescence (because of brain maturation). Young people that need help often have experienced very difficult situations.”*  (Professional, round 1, item 1.21)  *“Learning to do this* [communicating your opinion] *in a respectful way is very helpful I think. It has made me stronger.”*  (Young person, round 1, item 1.23) |
|  | Disagreement | *“In our service young people and older adults are mixed (25 to 65 years). We see that young people bring energy, a breath of fresh air and older people bring peace and quiet and social control…”*  (Professional, round 1, item 1.17)  *“I was still in high school when I got into AMHS. No school there. They didn’t even mention it once. Naturally a lot of people just quit in that case and don’t get their high school diploma. This has a huge impact on your life. The damage they can do is substantial, especially in that stage of life…”*  (Young person, focus group, item 2.19)  *“I think it can be beneficial to mix young people with older adults. They are at a different stage in life and can pass on their wisdom and experience.”*  (Young person, round 1, item 1.17)  *“Focus on the strengths of the person instead of putting them into a box by giving a diagnosis. Everything this person does afterwards will be associated with the diagnosis while this doesn’t always have to be ‘problematic’…”*  (Young person, round 1, item 2.10)  *“I learnt during dialectical behavior therapy that a diagnosis doesn’t mean the same for everyone. People with borderline are still different.”*  (Young person, round 3, item 2.10) |
| Hearing young people’s voices | Agreement | *“Sometimes taking a walk whilst talking to a healthcare provider can be helpful. The patient can express him-/herself better or can talk more easily because they don’t have to look directly at the healthcare provider.”*  (Young person, round 1, item 2.8)  *“A recreation room is also a good option. Not just for relaxation, but also to talk with a professional whilst doing a fun activity. I’ve noticed that a positive rapport between the young person and the professional is more easily established.”*  (Young person, round 1, item 1.15)  *“I had a healthcare professional whom I didn’t have a good connection with. However, I couldn’t and was not allowed to switch. This made our individual talks very challenging.”*  (Young person, round 2, item 1.33)  *“Since therapeutic alliance is an important condition for the efficacy of care, this* [switching healthcare professionals] *should be allowed. At the same time, it is important that young people don’t just go for the ‘popular’, less severe, or less confronting colleagues. Working through a tough relationship can be a healing and learning experience for young adults.”*  (Professional, round 2, item 1.33)  *“I think the young person should request it* [diagnostic testing]. *He/she/they will be able to judge whether a diagnosis would be helpful. Sufficient information should be provided so the young person can decide accordingly.”*  (Young person, round 2, item 2.10)  *“This* [being able to choose the format] *would make me feel more at ease because I am in control of how I want to communicate with the care providers.”*  (Young person, round 2, item 1.32) |
|  | Disagreement | *“There are a lot of young people that don’t dare to speak about the difficulties they encounter within a service. I think if there is a possibility to do this anonymously this might be helpful.”*  (Young person, round 1, item 1.35)  *“Youth participation and evaluation of the service is important to adjust the care provision if necessary. For example, in our service young people need to complete a survey at the end of their trajectory. We learned that they received too little information about medication. Since then, our psychiatrist has been giving monthly information sessions covering this topic.”*  (Professional, round 1, item 1.35)  *“Watch out for tokenism: if young people give feedback and nothing is done with it you need to tell them and explain why. Otherwise, this is useless…”*  (Young person, round 1, item 1.35)  *“Open communication is important. Why shouldn’t young people be allowed to hear what is said about them and read what is written about them?”*  (Young person, round 1, item 2.6)  *“This* [being able to read what healthcare providers write] *greatly increases the feeling of safety in young people. I think it also ensures that professionals remain as transparent and respectful as possible…”*  (Professional, round 1, item 2.6)  *“Maybe it’s good that everyone thinks carefully on what to write in a medical record. And you can still keep a notebook on the side in which you can write things down for yourself or one or two colleagues. Because that medical record sticks to the young person, what’s in it is in it.”*  (Parent, focus group, item 2.6)  *“On the one hand transparency is important, on the other hand the medical record is also a means of communication between healthcare providers which can be lost.”*  (Professional, round 1, item 2.6)  *“During my last admission, I ‘had’ to join the healthcare providers’ meeting at the end to get some feedback and get the chance to comment or add something myself. It gave me a lot of stress and up until this point I don’t really see the benefit of it (yet).”*  (Young person, round 1, item 2.12)  *“Young people should definitely be allowed to join meetings about them but there should also be meetings without them. Both are needed.”*  (Professional, round 1, item 2.12) |
| No man is an island | Agreement | *“Your environment certainly is important when you’re having a tough time. You cannot do it on your own. You need help from multiple sources.”*  (Young person, round 1, item 1.41)  *“Often a lack of connectedness is the main complaint. The goal should be to let them rely on their own network more to make support by professionals unnecessary.”*  (Professional, round 1, item 1.39)  *“Although the needs of the young person are important to focus on, attention should also be given to the needs of important others. If this cannot be taken up by the service itself, the service should collaborate with other services…”*  (Professional, round 1, item 1.36)  *“In child and adolescent psychiatry there are more consultations with the parents, real therapy sessions. And when you go to adult psychiatry…there your parents are allowed to come talk to the psychiatrist once a month. And you should specifically ask for it.”*  (Young person, focus group, item 2.18)  *“I think it’s useful to involve people in the immediate environment of the young person, with permission. They possibly have questions or have certain views and insights about the young person that could be of value for treatment. I think that it should go further than parents; people that the young person finds important and wants to see involved.”*  (Young person, round 2, item 2.18)  *“Keeping in touch with the outside world is super important to not become isolated.”*  (Young person, round 2, item 2.19)  *“This* [gradually preparing for the end of care] *is extremely important to make a successful transition to ‘normal’ life again. Unfortunately, this is almost never done. Although I was admitted several times, I’ve never gotten an achievable transition period.”*  (Young person, round 1, item 2.15)  *“There must be continuity of care, the care provision should not be abruptly terminated. Young people should know where to go (back to) if things are getting too hard.”*  (Parent, round 1, item 1.42)  *“I think it is sometimes easier to take things from someone that has also experienced it…than from an (unknown) professional.”*  (Young person, round 1, item 1.47)  *“I personally find it nice to have a conversation with someone who has experienced the same or similar things. Then you feel more like someone really understands you.”*  (Young person, round 1, item 1.47)  *“Assigning a buddy is a good idea, I think. In this way you can get to know someone on your first day in the service without it feeling awkward. They can make you familiar with the way things work on the ward.”*  (Young person, round 2, item 1.47)  *“The buddy is given responsibility and gets self-confidence. The new young adult gets to know someone right away and knows who to go to with practical questions.”*  (Young person, round 2, item 1.47) |
|  | Disagreement | *“—If the young person doesn’t want this* [parental involvement]*, this must be considered because not everyone has a good situation at home. Eventually, the young person knows what’s best for him-/herself in this situation. It can be harmful to then involve the immediate environment. Not only concerning trust in healthcare providers, but also in terms of the young person’s own trajectory.”*  (Young person, round 1, item 2.17)  *“My daughter got a Whatsapp message on New Year’s Eve from the crisis team. She was so happy because she hadn’t had contact with them in a long time. Just: Happy New Year…*  (Parent, focus group, item 2.16)  *“Multiple ways of communicating gives more options to young people so they can use what suits them best. Unfortunately, professionals want to stick with what they are used to: telephoning and meeting in person in the service.”*  (Professional, round 1, item 2.16)  *“There’s what’s ideal and what is possible. I like the idea* [of digital communication] *but my employer only wants to provide an outdated Nokia.”*  (Professional, round 1, item 2.16)  *“It takes a lot to become sufficiently resilient as a worker with experience to be able to take on a supporting role for young people. Not everyone is able to do this, even though they might really want to.”*  (Professional, round 1, item 2.20)  *“You should be careful with talking to other young people. In the facility I was in, specialized in eating disorders, everything was done in group; the professionals were just supervising a bit. It was impossible, young people were just trying to outdo each other.”*  (Young person, focus group, item 2.20) |
